# Knowledge, attitudes, and associated factors of caregivers towards children with autism spectrum disorder in East Coast Peninsular Malaysia

**DOI:** 10.7717/peerj.14919

**Published:** 2023-02-28

**Authors:** Nurkhaniza Kaman, Azlina Ishak, Juliawati Muhammad

**Affiliations:** Family Medicine Department, School of Medical Sciences, Health Campus, Universiti Sains Malaysia, Kubang Kerian, Kelantan, Malaysia

**Keywords:** Autism spectrum disorder, Caregiver, Knowledge on ASD, Attitude on ASD, Associated factor, ASD child

## Abstract

**Background:**

Caregivers are directly involved in managing the daily basic needs of children with autism spectrum disorder (ASD). The knowledge and attitudes of these caregivers are important to achieving success in their roles. Thus, this study aimed to determine what constitutes good knowledge, attitudes, and associated factors among caregivers toward children with ASD.

**Methods:**

A cross-sectional study was conducted among 128 caregivers of children with ASD in Kota Bharu, Kelantan from May to August 2020, using convenience sampling. Validated questionnaires were used to assess the knowledge and attitudes toward children with ASD. The data were analyzed using SPSS version 24. Descriptive statistics and simple and multiple logistic regression analyses were then performed.

**Results:**

The response rate was 100%. The proportion of good knowledge and attitudes toward children with ASD among caregivers was 85.1% and 88.3%, respectively. Factors significantly associated with good knowledge were being female (OR (95% CI) 2.79 [0.99–7.90]) and ASD children being non-first-born children (OR (95% CI) 0.41 [0.15–1.12]). Factors significantly associated with good attitudes were age of 30 years and older (OR (95% CI) 0.13 [0.03–0.62]) and caregiver having other children with other types of learning difficulties (OR (95% CI) 0.15 [0.04, 0.52]).

**Conclusions:**

The proportion of caregivers with good knowledge of ASD and good attitudes toward children with ASD was high. The caregiver’s age and sex, the position of the ASD child among the siblings, and the presence of other types of learning disorders in the family need to be considered when managing children with ASD.

## Introduction

Autism spectrum disorder (ASD) is a biological neurodevelopmental disorder that can be detected as early as 18 months old (Liu et al., 2016). According to the Diagnostic and Statistical Manual of Mental Disorders-5 (DSM-5), ASD comprises persistent deficits in social communication and interaction, which include deficits in social reciprocity, nonverbal communicative behaviors, and a lack of skills in developing, maintaining, and understanding relationships. Restricted and repetitive patterns of behavior, interests, or activities are also common in individuals with ASD (Lobar, 2016).

The prevalence of ASD shows an increasing trend in Asia and throughout the world, with male predominance at a ratio of 4:1 (Qiu et al., 2020). Thus, early identification of ASD in children is important, as this will improve the quality of life of patients and their families (Qiu et al., 2020). There are many available interventions to help children with ASD improve their living, communication and interaction skills (Ibrahim & Cronin, 2020). The success of these programs relies on the role played by the community and the caregivers of children with ASD.

Caregivers by definition are those who have the greatest involvement in patient care and assistance during the disease, and this role is critical to the patient’s adaptation and management of the condition (Mashayekhi, Pilevarzadeh & Rafati, 2015). Caregivers are directly involved in managing and handling children with ASD on a daily basis. Accordingly, on most days, they are often the first to encounter some of the distinctive behaviors shown by autistic children.

There have been many studies (Alharbi, 2018; Effatpanah et al., 2019; Luleci et al., 2016; Nur Adli et al., 2017; Rahbar, Ibrahim & Assassi, 2011; Salleh, Noor & Samsudin, 2018; White et al., 2019) that have examined the knowledge and attitudes toward children with ASD that were conducted among different populations who were either directly or indirectly involved in managing children with ASD. A study in Saudi Arabia has shown family and healthcare providers had good knowledge and attitudes toward ASD (Alharbi, 2018); however, a few studies have shown inadequate knowledge levels that need to be improved (Effatpanah et al., 2019; Luleci et al., 2016; Rahbar, Ibrahim & Assassi, 2011). Furthermore, two local studies have shown a wide range of knowledge levels among the general population (Nur Adli et al., 2017; Salleh, Noor & Samsudin, 2018).

In a related vein, a few studies (Anwar et al., 2018; Nur Adli et al., 2017; Russell, Steer & Golding, 2011) have highlighted the associated factors that significantly affect the knowledge and attitudes of caregivers toward children with ASD. Maternal age, maternal depression, and the position of the child among their siblings were shown to have a significant impact on the attitudes of caregivers (Russell, Steer & Golding, 2011), whereas another study found that females, those who were married, those who received tertiary educational levels, and those who earned more than RM 5000 were associated with a positive attitude toward ASD (Nur Adli et al., 2017). Anwar et al. (2018) concluded that females and the unmarried score significantly higher on both knowledge and attitudes toward ASD. Cultural background may also affect this knowledge and these attitudes, which will influence the interactions with the children. A study has shown that having had an interaction with someone with autism increases the chances of having another one (de Vries et al., 2020).

The general population might not be aware of ASD. This was reflected in a local study that showed that knowledge of ASD was still low despite the prevalence of good attitudes (Nur Adli et al., 2017). The level of knowledge and attitudes toward ASD in our setting are still lacking. As such, due to the varying knowledge and attitude levels worldwide, it is important to assess these domains among caregivers in our setting. As caregivers are directly involved with the care of ASD children, we hypothesize that their knowledge and attitudes will be found to be good. By identifying their level of knowledge, attitudes, and the factors affecting these domains, measures can be taken to strengthen the role of caregivers who support the daily lives of children with ASD. ASD children need help from caregivers to improve their living, communication, and interaction skills. Caregivers with good knowledge and attitudes will find more success in their roles in productive and positive interventions for children with ASD (Ibrahim & Cronin, 2020).

## Materials and Methods

This was a cross-sectional study conducted among 128 caregivers of children with ASD attending two tertiary hospitals in Kota Bharu, Kelantan, on the East Coast of Peninsular Malaysia between May 2020 and August 2020.

Inclusion criteria were persons who took care of and lived together with children with ASD who were aged 18 years and above and able to comprehend the Malay language, whereas those who were illiterate, had learning difficulties, or were deaf and mute were excluded. The sample size was calculated using a single proportion formula, with the prevalence of good knowledge of learning disorders having a value of 0.233 (Pawar & Mohite, 2014) and a precision of 0.08. The calculated sample size was 107, and after considering a 20% dropout rate, the total sample size was 128. Approximately 20 ASD patients were seen every month in each centre; thus, convenience sampling was applied.

Our research tool consisted of a new Malay validated questionnaire on knowledge of ASD and a newly developed and validated questionnaire on attitudes toward children with ASD. The questionnaire on knowledge of ASD was adapted from one developed by Anwar et al. (2018). The questionnaire underwent forward and backward translation from English to Malay by a group of panellists consisting of family medicine specialists, psychiatrists, and paediatricians who are bilinguals. Content validity was checked, and the revised version was tested on 40 caregivers of children with ASD for face validity. Reliability analysis was measured with a Cronbach’s alpha of 0.726. A Malay questionnaire on attitudes was designed following an extensive literature search. Expert judgement on the degree of relevance, representativeness for content validity, and degree of clarity was obtained from a group of experts, including a child psychiatrist, two paediatricians and two family medicine specialists. A comprehension for face validity was tested among 40 caregivers of children with ASD. Reliability analysis was measured with a Cronbach’s alpha of 0.755.

The questionnaire comprised three parts: (i) sociodemographic data, including child factors and medical-related factors; (ii) knowledge regarding ASD, consisting of 13 questions measured along a three-point Likert scale of “true”, “false” and “I don’t know”; (iii) attitudes of caregivers toward children with ASD, consisting of 25 questions (15 positive items and 10 negative items), with a four-point Likert scale ranging from “strongly agree”, “agree”, “disagree” and “strongly disagree”. For each knowledge question, a score of 1 was given if correctly answered, and 0 if wrongly answered or “I don’t know” was selected. The score for each question was summed up to obtain the total score, which ranged from 0 to 13. A score of 9 and above was considered good knowledge (Anwar et al., 2018). For the attitude questionnaire, “strongly agree”, “agree”, “disagree” and ‘strongly disagree’ (Russell, Steer & Golding, 2011) were each given a score of 4, 3, 2 and 1, respectively, for the positive attitude items. The scoring was reversed for the negative attitude items. The score for each question was added to obtain the total score, which ranged from 25 to 100. The expert theoretically assessed each item to determine the minimum score needed to be considered a good level of attitude. Then, the scores of all the items in the domain were summed and considered the cut-off point for that domain. In this questionnaire, the summation of the raw cut-off scores was converted to a percentage score, which gave rise to 75%. Therefore, those who scored more than 75% were considered to have a good attitude.

All caregivers of children with ASD attending the psychiatry clinics in both hospitals who fulfilled the inclusion and exclusion criteria were approached by the clinic staff. Brief information on the study was provided, and their contact numbers were obtained if they were keen to participate. The caregivers were approached again *via* a phone call by the researcher. Related information regarding the study was further explained, and written consent was obtained. The questionnaire was prepared in a Google form and was sent to the caregivers through email or WhatsApp applications. All questions were made compulsory, and submission was possible only if all questions had been attempted. The caregivers were given adequate time to answer and a reminder for the questionnaire’s completion and submission. The privacy and confidentiality of the participants and the data were respected at all times. The principles of this study and its operation were approved by the Human Research Ethics Committee, Universiti Sains Malaysia (IRB Reg No: 19080454), and the National Medical Research Registry (NMRR-19-2553-50057).

Data entry and analysis were performed using SPSS software version 24. The categorical variables were expressed as frequencies and percentages. Good knowledge and good attitudes were categorical variables; thus, logistic regression analysis was applied. A simple logistic regression (SLR) was used to screen for possible significant factors associated with good knowledge of ASD and good attitudes toward children with ASD. Factors with a *p-*value of less than 0.25 were further analyzed with a second analysis, using multiple logistic regression (MLR). This analysis was indicated as necessary to control for the confounding effect of the selected factors. The level of significance was set at *p* < 0.05. Meanwhile, the measurement of the association between the independent variables and the outcome was calculated by the odds ratio (OR) and 95% confidence interval (CI). The dependent variables were knowledge and attitudes, which were categorized into good or poor knowledge and good or poor attitudes, whereas the independent variables were age, sex, ethnicity, educational level, marital status, income, history of chronic illness, history of psychiatric illness, history of substance abuse, number of children in the family, the position of the children with ASD among the siblings, presence of other children with ASD and presence of other children with other types of learning disorders.

## Results

All 128 caregivers participated in the study, leading to a response rate of 100%.

The majority of the caregivers were female (68.7%), Malay (97.7%) and married (96.9%). The details of the sociodemographic profiles of the caregivers are shown in Table 1.

**Table 1:** Sociodemographic characteristic of caregivers.

Variables	*n* (%)
Age <30 ≥30	7 (5.5) 121 (94.5)
Educational level Secondary Tertiary	35 (27.3) 93 (72.7)
Income <RM 1,000 ≥RM 1,000	29 (22.7) 99 (77.3)
History of chronic illness Yes No	5 (3.9) 123 (96.1)
History of psychiatric illness Yes No	1 (0.8) 127 (99.2)
History of substance abuse Yes No	0 128 (100)
No of children in family 1 >1	7 (5.5) 121 (94.5)
Position of ASD child 1^st^ born Not 1^st^-born	71 (55.5) 57 (45.5)
Other children with ASD Yes No	13 (10.2) 115 (89.8)
Other children with other type of learning disorders Yes No	19 (14.8) 109 (85.2)

### Proportion of good knowledge and its associated factors among caregivers

Most of the caregivers had good knowledge of ASD (85.1%). Factors associated with good knowledge of ASD that were determined through SLR and MLR are shown in Tables 2 and 3. In the sociodemographic data, out of 13 variables, we noted small cell categories between the groups for ethnicity (125 *versus* three), history of psychiatric illness (1 *versus* 127) and history of substance abuse (zero *versus* 128), thus, these three factors were not suitable for univariable and multivariable analysis. Therefore, we included only 10 out of the 13 variables in the SLR. From the SLR analysis, sex (*p* = 0.11), educational level (*p* = 0.07), and position of ASD child (*p* = 0.03) were included in the MLR. From the MLR analysis, only two factors were shown to be significant, with a *p*-value < 0.05, and these were displayed in the MLR table. The MLR showed that being female and having a child with ASD as a non-first-born child were significantly associated with good knowledge about ASD among caregivers. It was shown that women had 2.79 times higher odds of having good knowledge of ASD compared to men (95% CI [0.99–7.99]). Caregivers who had a child with ASD who was not the first-born showed 59% lower odds of having good knowledge of ASD (95% CI [0.15–1.12]) as compared to caregivers who had a first child with ASD.

**Table 2:** Simple logistic regression for good knowledge.

Variables	Crude *OR* (95% CI)	Wald stat	*p*-value
Age <30 ≥30	1 1.16 [0.20–4.63]	0.04	0.83
Sex Male Female	1 2.27 [0.84–6.11]	2.61	0.11
Educational level Secondary Tertiary	1 2.33 [0.92–5.91]	3.19	0.07
Marital status Married Single/Divorced	1 0.51 [0.05–5.17]	0.33	0.57
Income <RM 1,000 ≥RM 1,000	1 1.10 [0.55–2.20]	0.07	0.79
History of chronic illness No Yes	1 0.69 [0.07–6.49]	0.11	0.74
No of children in family 1 >1	1 0.95 [0.11–8.40]	0.01	0.97
Position of ASD child 1^st^ born Not 1^st^-born	1 0.41 [0.15–1.12]	3.00	0.03
Other children with ASD No Yes	1 0.95 [0.19–4.69]	0.01	0.95
Other children with other type of learning disorders No Yes	1 1.57 [0.33–7.43]	0.32	0.57

**Table 3:** Multiple logistic regression for good knowledge.

Variables	Adjusted *OR* (95% CI)	Wald stat	*p* value
Sex Male Female	1 2.79 [0.99–7.99]	3.75	0.04
Position of ASD child 1^st^ born Not 1^st^ born	1 0.41 [0.15–1.12]	3.00	0.03

### Proportion of good attitudes and its associated factors among caregivers

Most of the caregivers had a good attitude toward their children with ASD (88.3%). Factors associated with a good attitude toward children with ASD, as determined by SLR and MLR are shown in Tables 4 and 5. MLR showed that the age of the caregivers and the presence of other children with other types of learning disorders were found to be significantly associated with a good attitude toward children with ASD. Caregivers who were older than 30 years of age had 87% lower odds of having a good attitude toward ASD children (95% CI [0.03–0.62]). Finally, caregivers who had other children with other types of learning disorders had 85% lower odds of having a good attitude toward children with ASD (95% CI [0.04–0.52]) compared to caregivers who did not have other children with learning disorders.

**Table 4:** Simple logistic regression for good attitude.

Variable	Crude OR (95% CI)	Wald stat	*p* value
Age ≤30 >30	1 0.21 [0.05–0.87]	4.61	0.03
Sex Male Female	1 2.12 [0.71–6.32]	1.82	0.18
Educational level Secondary Tertiary	1 1.21 [0.44–3.34]	0.14	0.71
Marital status Married Single/Divorced	1 2.22 [0.50–5.20]	0.40	0.90
Income <RM 1,000 >RM 1,000	1 1.28 [0.59–2.76]	0.39	0.54
History of chronic illness No Yes	1 0.18 [0.03–1.16]	3.26	0.07
No of children in family 1 >1	1 1.27 [0.14–11.37]	0.05	0.83
Position of ASD child 1^st^ born Not 1^st^-born	1 0.49 [0.16–1.48]	1.60	0.21
Other children with ASD No Yes	1 0.70 [0.14–3.52]	0.19	0.67
Other children with other type of learning disorders No Yes	1 0.20 [0.06–0.64]	7.33	0.01

**Table 5:** Multiple logistic regression for good attitude.

Variables	Adjusted *OR* (95% CI)	Wald stat	*p* value
Age ≤30 >30	1 0.13 [0.03–0.62]	6.51	0.01
Other children with other type of Learning disorders No Yes	1 0.15 [0.04–0.52]	8.85	0.003

## Discussion

This study aimed to explore caregivers’ knowledge and attitudes toward children with ASD in Malaysia, as they are the closest group dealing with these special children. A comprehensive assessment of the knowledge and attitudes of the caregivers was conducted through the administration of a validated questionnaire.

Our study concluded that the majority of the caregivers from Kota Bharu, Malaysia had good knowledge of ASD. This is comparable to a study done by Alharbi (2018) among the family members of ASD children. In contrast, a study by Anwar in Pakistan showed that parents still had poor knowledge of ASD (Anwar et al., 2018). ASD is still a relatively undiscussed disorder among the Pakistani population, and they remain completely unaware of the condition. Meanwhile, two local studies also showed poor knowledge of ASD. These studies were conducted among the general population, who might not be fully aware of the presence of the disorder (Nur Adli et al., 2017; Salleh, Noor & Samsudin, 2018). Our study focused on the caregivers of children with ASD, as they have continuous and daily exposure to the children, so it is easier for them to recognize the characteristics and behavior of children with ASD.

Interestingly, our study found that females were more prone to having good knowledge of ASD than men, and this finding was similar to a study done in Pakistan (Anwar et al., 2018). Anwar et al. (2018) reported that women had a statistically significantly higher score for opinions, and signs and symptoms of autism. A study in France also reported that women showed greater awareness and a better understanding of the specific characteristics of autism, schizophrenia and bipolar disorders when compared to men (Durand-Zaleski et al., 2012). As men and women often have different roles in taking care of their children, (Campaña, Giménez & Molina, 2015), some studies have indicated that women tend to demonstrate more positive attitudes toward autism (Jensen et al., 2015; Kuzminski et al., 2019). By being more involved in the care of their children, they tended to be more observant of their children’s behaviour. Furthermore, women are known to have better knowledge acquisition skills than men (Evans, Schweingruber & Stevenson, 2002) which further explains why they have better knowledge of ASD compared to men.

Our study also found that the position of children with ASD in the family was an important determinant of having good knowledge among caregivers. Having a first child with ASD is likely to trigger more proactive emotions and behaviors toward learning and seeking new knowledge and experience among inexperienced caregivers, compared to having subsequent children with ASD. As the number of children increases, caregivers become more stressed (Qian et al., 2020), and thus less attention would be paid to caretaking. The caregivers’ time would then be consumed mainly by other activities, rather than by finding information about the disease.

Caregivers in our study population had good attitudes toward children with ASD, and this finding was similar to a local study done among the general population (Nur Adli et al., 2017). This finding was further supported by a study in Pakistan, which also showed that parents had a high level of positive attitudes toward children with ASD (Anwar et al., 2018).

Caregivers aged more than 30 years were less likely to have a good attitude. Being an older mother has been shown to contribute to an increased stress level,(Duarte et al., 2005) which would affects their attitude toward ASD. However, two studies (Al-Oran & AL-Sagarat, 2016; Russell, Steer & Golding, 2011) had findings that varied from ours. Russell, Steer & Golding (2011) concluded that older mothers were more experienced, good at identifying their child’s odd behaviour, and more confident when bringing their children for a medical check-up. Al-Oran & AL-Sagarat (2016) stated that increasing maternal age was associated with a good attitude and good stress management while handling children with ASD.

Caregivers who had children with other types of learning difficulties were less likely to have a good attitude toward children with ASD. Mothers who already have children with ASD are at a higher risk of stress (Duarte et al., 2005). It has also been shown that parents of children with ASD have a stress level that is much higher compared to parents who have children with normal development (Giovagnoli et al., 2015). Having more children with learning difficulties predisposes caregivers to have more emotional disturbances, which adds to their frustration and poses higher risks of depression. They might also put the blame on themselves for what had happened to their children. In another study, mothers who had more than one child with learning disorders carried high levels of depression and low quality of life compared to mothers with children without learning disorders (Al-Oran & AL-Sagarat, 2016). Furthermore, maternal depression may act as an “access barrier” to clinical intervention and thus prevent them from seeking clinical help for their children (Russell, Steer & Golding, 2011).

Our study did have some limitations. This study was conducted *via* the WhatsApp application; thus, the knowledge of the participants might not be accurate, as they might have been influenced by other people when filling out the questionnaires. In addition, our study population was mainly of Malay ethnicity; thus, generalizability to other populations should be interpreted with this in mind.

## Conclusions

A good level of knowledge and good attitudes among the caregivers in our study reflected a good care offered toward this special group of children. Other significant and non-modifiable associated factors found in this study that affect the knowledge and attitude of caregivers need to be discussed with caregivers during the early management of their children. Further research regarding the current educational opportunities for school-going children with ASD needs to be carried out. Giving them a fair chance to participate in and get involved in all levels of education similar to other children until the tertiary educational level is also part of exhibiting a good attitude towards children with ASD.

## Supplemental Information

10.7717/peerj.14919/supp-1 Supplemental Information 1 Raw data.

## Additional Information and Declarations

### Field Study Permissions

The following information was supplied relating to field study approvals (*i.e*., approving body and any reference numbers): Field experiment was approved by National Medical Research Register (NMRR) Malaysia (NMRR-19-2553-50057).

### Data Availability

The following information was supplied regarding data availability: The raw data is available in the Supplemental File.
